# Biomimetic Anticoagulated Porous Particles with Self‐Reporting Structural Colors

**DOI:** 10.1002/advs.202400189

**Published:** 2024-03-23

**Authors:** Hanxu Chen, Feika Bian, Zhiqiang Luo, Yuanjin Zhao

**Affiliations:** ^1^ Department of Rheumatology and Immunology Nanjing Drum Tower Hospital School of Biological Science and Medical Engineering Southeast University Nanjing 210096 China; ^2^ Shenzhen Research Institute Southeast University Shenzhen 518038 China; ^3^ Chemistry and Biomedicine Innovation Center Nanjing University Nanjing 210023 China

**Keywords:** anticoagulation, bioinspired, hydrogel, pattern recognition, structural color

## Abstract

Anticoagulation is vital to maintain blood fluidic status and physiological functions in the field of clinical blood‐related procedures. Here, novel biomimetic anticoagulated porous inverse opal hydrogel particles is presented as anticoagulant bearing dynamic screening capability. The inverse opal hydrogel particles possess abundant sulfonic and carboxyl groups, which serve as binding sites with multiple coagulation factors and inhibit the blood coagulation process. Owing to the variations of refractive index and pore sizes during the binding process, the particles appeared corresponding structure color variations, which can be adopted as sensory index of anticoagulation. Based on these features, a sensor containing these diverse structure color particle units is constructed for pattern recognition of coagulation factors level in clinical plasma samples. By analyzing the sensory information of the unit, the colorimetric “fingerprint” for each target can be obtained and summarized as a database. Besides, a portable test‐strip integrating sensory units is developed to distinguish the sample regarding abnormal coagulation factors‐derived diseases via multivariate data analysis. It is believed that such biomimetic anticoagulated structural color particles and their derived sensor will open new avenue for clinical detection and disease diagnosis.

## Graphical abstract

Biomimetic anticoagulated porous particles with inverse opal hydrogel structures and self‐reporting structural colors for anticoagulation screening are proposed. The characterized structural color variations of particles during binding with coagulation factors are adopted for sensory. Additionally, a pattern‐recognition‐based particle‐integrated sensor is constructed with great potential for point‐of‐care‐testing (POCT) applications and clinical disease diagnosis.

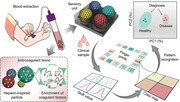

## 1 Introduction

Blood anticoagulation has aroused extensive attention for clinical applications, including hemodialysis, hemoperfusion, blood storage, blood routine examination, and so on.^[^ ^1^, ^2^, ^3^, ^4^, ^5^, ^6^, ^7^ ^]^ With the aim to prevent the formation of blood clots and remain fluid status, a series of anticoagulants have been developed to achieve efficient inactivation of blood coagulation, such as sodium citrate, ethylenediaminetetraacetic acid disodium salt, heparin, etc.^[^ ^8^, ^9^, ^10^, ^11^ ^]^ As a representative natural anticoagulant medication, heparin appeared binding affinity and inhibiting effects to certain coagulation factors owing to highly sulfated and carboxylated chemical structure. Benefitting from its capability of disturbing endogenous and exogenous clotting pathways, heparin and its derivations have thus achieved extensive applications in the fields of systemic anticoagulation, anti‐inflammation, angiogenic treatment, etc.^[^ ^12^, ^13^, ^14^, ^15^, ^16^, ^17^, ^18^, ^19^ ^]^ However, due to the lack of dynamic evaluation stratagem, traditional heparin anticoagulants still suffer from the problems of excessive dosage, and the incomplete reversal of heparin within blood could lead to excessive bleeding risks and interference of blood analysts.^[^ ^20^, ^21^, ^22^ ^]^ In addition, as these heparins and derivations are from animal sources, their biosafety problems of potential virus contamination and adverse effects deserve more attention.^[^ ^23^, ^24^, ^25^ ^]^ Therefore, new anticoagulation strategy bearing abilities of biosafety and real‐time screening are still highly anticipated.

**Figure 1:**
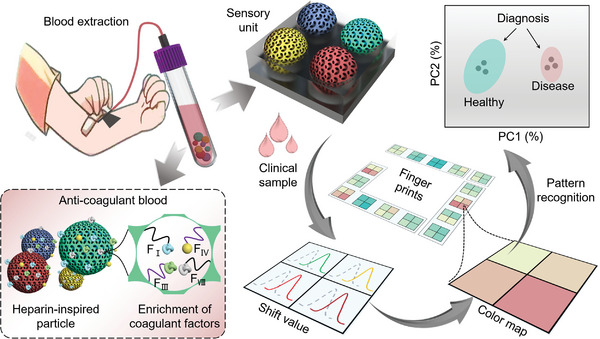
Schematic diagram of the biomimetic porous particles with self‐reporting structural colors for anticoagulation screening. The anticoagulation function was achieved due to the adsorption of several coagulant factors. The diagnosis of healthy and disease states was based on the pattern recognition of the shift value database (fingerprints) containing structural color variations.

Here, we developed novel biomimetic anticoagulated porous particles with inverse opal hydrogel structures and self‐reporting structural colors for anticoagulation screening, as schemed in **Figure** **1**. Photonic crystals (PhCs) are materials bearing spatial periodicity of dielectric constant, and modulating electromagnetic waves with sufficient dielectric contrast and appropriate geometry. As a representative photonic crystal (PhC) material, the inverse opal is featured with highly ordered hexagonal close packing and interconnected cavity structures.^[^ ^26^, ^27^, ^28^, ^29^, ^30^, ^31^ ^]^ The sufficient specific surface area of the inverse opal hydrogel skeleton provided abundant binding sites between functional groups (sulfonic and carboxyl) and multiple coagulation factors. Such chemical anchoring of the anticoagulation reagents will not only achieve extracorporeal anticoagulation of blood but also avoid the risk of residuals in the blood and improve the biosafety. Additionally, due to the photonic band gap (PBG) derived from the intrinsic hexagonal close‐packed lattice, only light bearing specific wavelengths could be reflected, thus inverse opal particles were imparted with iridescent structural colors.^[^ ^32^, ^33^, ^34^, ^35^, ^36^ ^]^ During the above binding process, the refractive index and pore sizes of particles were altered, meanwhile, the shift of characteristic reflection peak values and structural color were observed with respective variations. Thus, it was conceived that such inverse opal particles could be adopted as intelligent elements for anticoagulation with a dynamic screening process.

In this paper, we constructed the desired anticoagulated inverse opal hydrogel particles and employed them as sensory units with diverse structure colors for pattern recognition of multiple coagulation factors (Figure 1). By analyzing the shift values of each sensory unit, a colorimetric “fingerprint” for each target could be obtained and stored in a database. Besides, a portable test‐strip integrated with our sensory unit was proposed for convenient monitoring of clinical plasma samples, and the diseases derived from abnormal coagulation factors could be detected according to multivariate data analysis. In addition, the blood safety of the particles and the reusability of the device were examined, which indicated great potential for practical clinical applications. These features make such biomimetic anticoagulated porous self‐reporting structural color particles highly promising for point‐of‐care‐testing (POCT) applications and clinical disease diagnosis.

## 2 Results and Discussion

**Figure 2:**
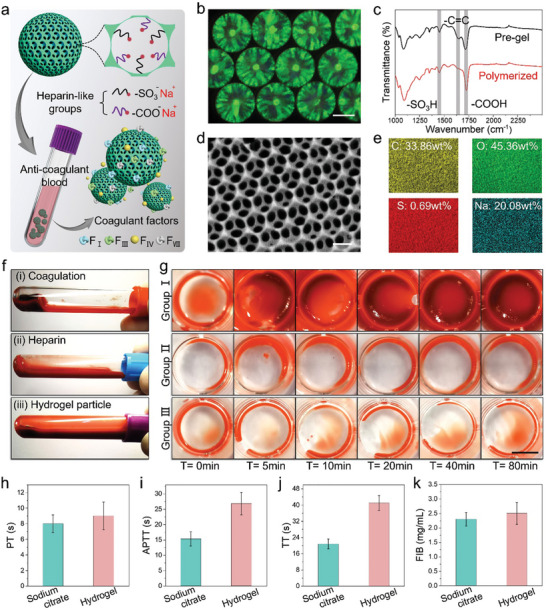
Characterization of anticoagulation property of SCIOPs. a) Schematic diagram of the anticoagulation mechanism of absorbing certain coagulation factors by SCIOPs. b) The reflection image of SCIOPs. c) The FT‐IR spectrum of anticoagulated hydrogel before and after polymerization. The characteristic peaks of carboxyl group (1726.46 cm^−1^) and sulfonic group (1450.69 cm^−1^) were labeed with gray. The C═C peak (1639.19 cm^−1^) disappeared after polymerization. d) The SEM image of the inverse opal structures of SCIOPs. e) The maps and quantitative contents of four main elements of SCIOPs (C, O, S, Na). f) The optical images of fresh blood treated with (i) none anticoagulation, (ii) heparin, and (iii) SCIOPs. g) The WBCT images of fresh blood incubated within three groups at different times. h–k) Comparison of four blood coagulation indexes between sodium citrate and hydrogel particles. The scale bars are 250 µm in (b), 250 nm in (d), and 4 mm in (g).

In a typical experiment, the structural color inverse‐opal particles (SCIOPs) were fabricated by replicating the topological structure of PhCs templates (Figure S1a, Supporting Information). As previously reported, the PhCs templates could be obtained via confined self‐assembly of silicon dioxide nanoparticles in the microfluidic droplets (Figure S1b, Supporting Information). Subsequently, the PhCs templates were immersed in the pre‐gel solution until the spaces between nanoparticles were totally infiltrated. After polymerization triggered by UV‐light (Figure S1c, Supporting Information), the templates were finally etched and hydrogel inverse opal skeletons remained, namely SCIOPs. Such pre‐gel solution was comprised of two main functional components, including the hydrogel skeleton (polyethylene glycol diacrylate (PEGDA)) and the anticoagulation unit. In detail, the anticoagulation unit was featured with heparin‐mimic functional groups like carboxyl and sulfonic groups bearing affinity to certain coagulation factors (**Figure** **2**a), where the former was provided by acrylic acid (AA), and the latter was integrated by 2‐acrylamido‐2‐methyl‐1‐propanesulfonic acid (AMPS). To enhance their anticoagulation property, the SCIOPs were treated with sodium hydroxide (NaOH) to form sodium salt (─COONa and ─SO_3_Na), and the optical image of the final SCIOPs was shown in Figure 2b. Especially, the Fourier transform infrared spectroscopy (FT‐IR) was measured to explore the crosslinking process and confirm the successful integration of anticoagulation groups (Figure 2c). The cross‐linking process was proved to occur via a carbon‐carbon double bond (C═C) whose peak disappeared after polymerization (1639.19 cm^−1^), and the peaks associated with the carboxyl group (1726.46 cm^−1^) and sulfonic group (1450.69 cm^−1^) invariably remained. The peaks of carboxyl group and sulfonic group were also proved to be consistent with the heparin sodium (Figure S2, Supporting Information).

The integration of such two functional groups mainly aimed to mimic the anticoagulation mechanism of heparin, which combined with antithrombin for catalysis of the coagulation factor inhibition. The binding sites between hydrogel and numerous biomolecules were efficiently inactivated by the subsequent formation of ─COONa and ─SO_3_Na, rather than activating the intrinsic pathway of the coagulation cascade. Besides, the affinity to several coagulation factors also contributed to the anticoagulant performance. The concentration of antithrombin‐III (AT‐III) in the blood treated with heparin and SCIOPs was measured, and the results showed the anticoagulant hydrogel materials possessed similar interactions with antithrombin compared with heparin (Figure S3a, Supporting Information).^[^ ^3^ ^]^ We further examined the activity of FXa in the plasma treated with SCIOPs. A clear decrease in FXa activity indicated the prohibition of the FXa‐thrombin which was induced by heparin or heparin‐mimicking SCIOPs (Figure S3b, Supporting Information). Similarly, the concentration of FXIIa in heparin and SCIOPs‐treated plasma samples was also detected in Figure S3c (Supporting Information). The results revealed the inhibitor role of SCIOPs like heparin on the intrinsic coagulation pathways. A scanning electron microscope (SEM) was adopted to observe the inverse opal structures of SCIOPs (Figure 2d). Due to the highly ordered pores, SCIOPs were endowed with abundant specific surface areas for the adsorption of coagulation factors. Besides, the energy dispersive spectrometer (EDS) analysis was performed to detect the elements distribution of SCIOPs (Figure 2e; Figure S4, Supporting Information).

It has been demonstrated that the hydrophilic property of SCIOPs contributes to the full contact and affinity with blood components. As presented in Figure S5a–d (Supporting Information), the water contact angle of the hydrogel components of SCIOPs was examined (including PEGDA, PEGDA/AA, PEGDA/AMPS and PEGDA/AA/AMPS). After treatment with NaOH, the hydrophilic property was further improved (Figure S5e, Supporting Information), which better signified the improved compatibility between SCIOPs and blood components. Then the anticoagulation property of SCIOPs was investigated, where three experimental groups were established including a non‐anticoagulation tube, heparin‐coated tube, and SCIOPs‐contained tube. The coagulation blood clots were obviously observed in non‐anticoagulation tube, while no blood clots formed in heparin‐coated tube and SCIOPs‐contained tube (Figure 2f; Figure S6, Supporting Information). In addition, the whole blood clotting time (WBCT) of three groups was measured in Figure 2g and the optical images of blood clotting were recorded at different times. The blood treated with SCIOPs was observed without clots even after 80 min, thus the blood could be thought as totally anticoagulated. To further validate the anticoagulation property of SCIOPs, four blood coagulation indexes including prothrombin time (PT), activated partial thromboplastin time (APTT), thrombin time (TT), and concentration of fibrinogen in the blood (FIB) were assessed, comparing hydrogel particles with typical anticoagulation (Figure 2h–k). In particular, the APTT and TT values of fresh blood were obviously prolonged, due to the abundant adsorption of certain coagulation factors. These features indicate that the SCIOPs possessed superior anticoagulation properties.

**Figure 3:**
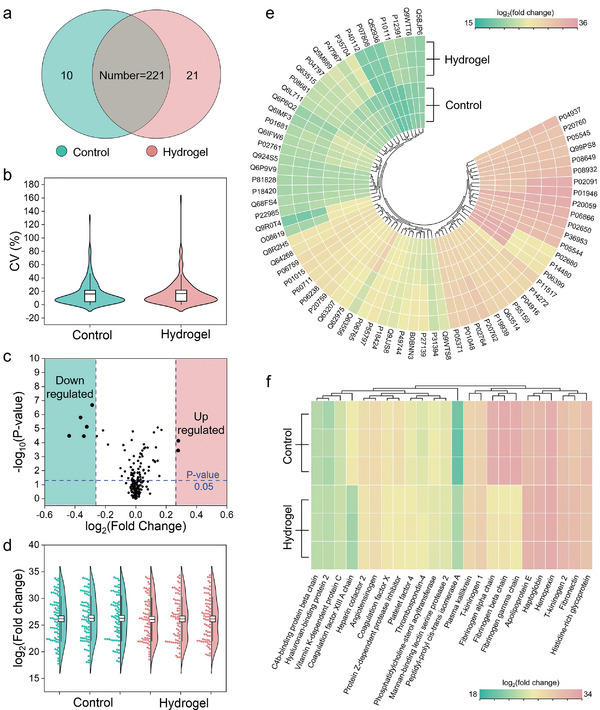
Proteomics analysis of the plasma samples separated from the whole blood treated with heparin (control) and SCIOPs (hydrogel). a) The Venn plot of the protein species of two groups. b) The CV value of two groups. c) The volcano plot of the protein abundance of hydrogel groups compared with control groups. d) The violin plot and colony chart of three parallel samples of two groups. e) The polar heat‐map of the protein abundance of two groups. f) The heat‐map of the abundance of anticoagulation‐related proteins of two groups.

Furthermore, proteomics analysis of the plasma samples was performed to investigate the variations in protein species and their abundance. The plasma separated from whole blood with heparin and hydrogel SCIOPs served as control and experimental groups, respectively. As presented in **Figure** **3**a, it was found that there existed a subtle distinction of protein species between the two groups. Thus, the direct contact of SCIOPs with whole blood applied few damages to the functional protein components when compared to the standard blood anticoagulation procedure. First, the coefficient of variation (CV%) of the two groups was tested to prove the validity of plasma samples during analysis of biological mass spectrometry (Figure 3b). Then the difference between the abundance of proteins is investigated in Figure 3c. It was found that only two proteins were up‐regulated and five proteins were down‐regulated, while the other proteins remained at similar abundance level. Among the five down‐regulated proteins, the abundance of fibrinogen alpha, beta, and gamma chain were lower than control group, which further confirmed the adsorption of fibrinogen by SCIOPs during the anticoagulation process. The abundance of each three parallel samples of control group and experimental group is shown in Figure 3d. Besides, the polar heat‐map was adopted to better display the difference of protein abundance between the two groups (Figure 3e; Table S1, Supporting Information). A logarithmic representation was adopted to show the differences more clearly. The red color means proteins with higher abundance in the samples, and the green color represents proteins with lower abundance. Among these proteins, the proteins related to the anticoagulation mechanism were paid with more attention, and their heat‐map of abundance with cluster was summarized in Figure 3f. Apart from the down‐regulation of three fibrinogen chains, some other proteins, especially heparin cofactor2, were observed with similar abundance between the two groups, which indicated the analogous functions of SCIOPs for anticoagulation compared with heparin.

As mentioned above, the SCIOPs consisted of abundant nanopores arranged in a hexagonal close‐packing configuration. Such periodic highly‐ordered structures endowed SCIOPs with brilliant structural colors. According to the Bragg's equation:

(1) $$\lambda \;=\;1.633{\mathrm{dn}}_{\mathrm{average}}$$

where the λ is the characteristic reflection peak, d is the diameter between two neighboring nanopores, n_average_ is the average refractive index of the hydrogel components. It can be deduced from this equation that the position of λ can be optimized by adjusting the nanopores diameter and the average refractive index. Benefitting from the abundant carboxyl and sulfonic groups, the SCIOPs exhibited strong affinity to certain coagulation factors, including fibrinogen (F_I_), thromboplastin (F_III_), calcium ion (F_IV_), and globulin antihemophilia (F_VIII_). The affinity to proteins of SCIOPs treated with NaOH was demonstrated to be weak due to the occupation of active binding sites of the functional groups and increased hydrophilia. The lower adsorption of typical plasma protein (bovine serum albumin) contributed to the reduction of albumin loss and eliminated the interference during the sensing and anticoagulant process (Figure S7, Supporting Information), which also weakened the interaction with fibrinogen.

**Figure 4:**
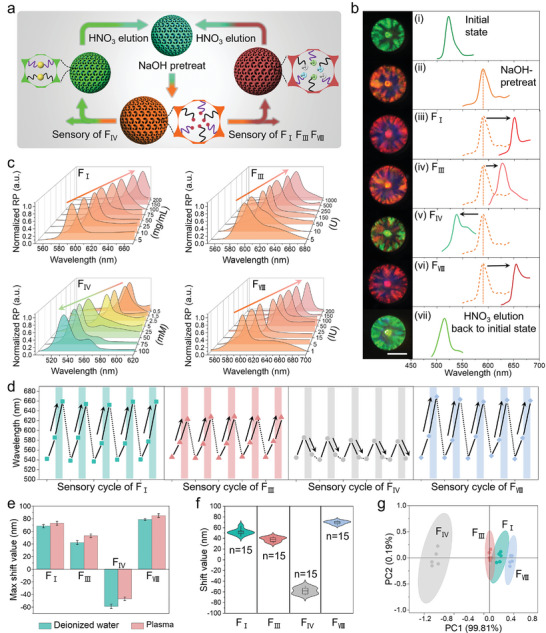
Sensory procedure of four coagulation factors by SCIOPs. a) Schematic illustration of the sensory mechanism of F_I_, F_III_, F_IV_ and F_VIII_. b) The optical images and reflection spectra of SCIOPs during the whole sensory procedure. c) The reflection spectra of SCIOPs immersed in four coagulation factor solutions with different concentrations. d) The shift values of SCIOPs during five sensory recycles of four coagulation factors. e) The comparison of shift values of four coagulation factors in deionized water and plasma. f) The database of shift values of F_I_, F_III_, F_IV,_ and F_VIII_ based on fifteen SCIOPs. g) The PCA score plot of shift values of four coagulation factors. The scale bar is 250 µm in (b).

During the adsorption process of different targets, the characteristic reflection peaks of SCIOPs were observed with corresponding red shift (F_I_, F_III_, and F_VIII_) and blue shift (F_IV_), as illustrated in **Figure** **4**a. The red shifts were the consequence of the increment of the respective average refractive index after the adsorption of three coagulation factors, while the blue shift was caused by the decrease of nanopores diameter due to the chelation interaction between carboxyl groups with F_IV_. It was worth mentioning that the variations of shift values were proved to be related to the concentration level of these four targets. Such relationships could be utilized as the sensory principle to monitor the adsorption process (Figure 4c). For better combination with F_IV_, the SCIOPs were first treated with NaOH to form ─COO^−^Na^+^, which was subsequently replaced by F_IV_ and formed coordinate covalent bonds (─COOH─Ca─COOH). Then the variations of reflection wavelength of SCIOPs during pretreatment (Figure 4b i,ii) and adsorption processes (Figure 4b iii–vi) were recorded. Besides, it was found that nitric acid (HNO_3_) was able to efficiently elute the four coagulation factors, together with the recovery of the initial structural color of SCIOPs (Figure 4b vii).

Thus, a whole sensory recycle was established with three steps, including NaOH treatment, adsorption of coagulation factors, and HNO_3_ elution. Five sensory recycles for each target were repeated, and the results showed excellent stability which imparted SCIOPs with great potential for recyclable use in practical applications (Figure 4d). In addition, a comparison of the maximum shift values for four coagulation factors was conducted in deionized water and plasma (Figure 4e). It was found that the shift values are relatively larger in plasma because of the inevitable swelling of SCIOPs. Certainly, the swelling phenomenon was relatively too slight and the derived interference on shift values was negligible. Furthermore, considering of the specific shift directions and values of characteristic reflection peaks, the pattern recognition method was utilized to distinguish four coagulation factors. The shift values of four coagulation factors were measured by fifteen SCIOPs, respectively (Figure 4f). The principal component analysis (PCA) was performed based on the database of shift values, and the results showed a distinct separation of four coagulation factors (Figure 4g).

**Figure 5:**
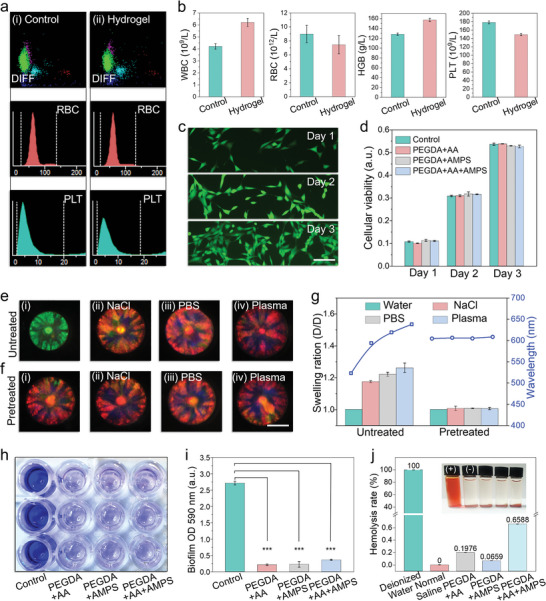
Characterization of biosafety of SCIOPs. a) The DIFF, RBC, and PLT volume distribution of whole blood was incubated with (i) EDTA‐K_2_ and (ii) SCIOPs. b) The blood routine test results including WBC, RBC, HGB, and PLT of whole blood treated with EDTA‐K_2_ and SCIOPs. c) The optical images of NIH‐3T3 cells cultured with SCIOPs for 3 days. d) The viability of cells cultured with multiwell plate, PEGDA/AA, PEGDA/AMPS, and PEGDA/AA/AMPS for 3 days. e,f) The reflection images of untreated particles and NaOH‐pretreated SCIOPs in deionized water, NaCl, PBS, and plasma solutions. g) The swelling ratio and characteristic wavelength of initial particles and NaOH‐pretreated SCIOPs. h) The optical images of staining of crystal violet of an agar plate, PEGDA/AA, PEGDA/AMPS, and PEGDA/AA/AMPS. i) The optical density (OD) intensity of biofilm of *S. aureus* at 590 nm. j) The hemolysis ratio of SCIOPs. The inner box is the optical images of RBCs incubated with distilled water (+), normal saline (−), and hydrogel components of SCIOPs. The scale bars are 80 µm in (c) and 200 µm in (f).

Due to the direct contact between SCIOPs and fresh whole blood, the biosafety of anticoagulated hydrogel components should be guaranteed. The whole blood samples were incubated with ethylene diamine tetraacetic acid‐K_2_ (EDTA‐K_2_) and SCIOPs, respectively. Subsequently, differential white blood cell count (DIFF) scatter charts, red blood cell (RBC), and platelet (PLT) volume distribution were examined, as presented in **Figure** **5**a. The blood routine test results including white blood cell (WBC), RBC, hemoglobin (HGB), and PLT (Figure 5b) were also assessed. Besides, the complement fragment 3a (C3a) and complement fragment 5a (C5a) were chosen for evaluation of the influence on the complement activation system (Figure S8, Supporting Information). The concentration of thrombin‐antithrombin (TAT) and bradykinin (BK) were examined to optimize the effects of SCIOPs on contact activation system and kallikrein‐kinin system (Figure S9, Supporting Information). These comprehensive analyses demonstrated that SCIOPs exhibited negligible impact on blood cells and appeared reliable biosafety. In addition, the NIH‐3T3 cells were cultured with the hydrogel components of SCIOPs, and the biocompatibility was explored based on 3‐(4,5)‐dimethylthiahiazo(‐z‐yl)−3,5‐di‐phenytetrazoliumromide (MTT) assay for 3 days (Figure 5c,d). The different combinations of hydrogel components (PEGDA/AA, PEGDA/AMPS and PEGDA/AA/AMPS) were all proved to bear analogous biocompatibility with cells compared with commercial multi‐well plates.

It was found that the initial untreated SCIOPs swelled obviously with red shift of reflection wavelength. To mitigate the interference of sensory caused by swelling in practical clinical samples, the SCIOPs were pretreated with NaOH solutions. In contrast, the SCIOPs treated with NaOH showed anti‐swelling properties in sodium chloride solution (NaCl), phosphate buffer solution (PBS), and plasma samples (Figure 5e–g; Figure S10, Supporting Information), which represented common physiological liquid environments. Besides, an evaluation of antibacterial adhesion was deemed imperative for clinical applications, due to the potential risk of infection or cross‐contamination of the biological samples. In this condition, the hydrogel components were divided into three experimental groups (PEGDA/AA, PEGDA/AMPS, and PEGDA/AA/AMPS), with an agar plate serving as the control group. Staphylococcus aureus (*S. aureus*) was selected as the model bacteria to be cultured with th above four groups. As shown in Figure 5h,i, the optical images of crystal violet staining proved that the bacteria could not survive on the surface of hydrogel components of SCIOPs, which was due to the destruction effect on the biofilm.

The blood compatibility of SCIOPs was also analyzed by measuring the hemolysis ratio (Figure 5j). It was observed that the hydrogel components of SCIOPs caused no hemolysis of RBCs. In brief, the SCIOPs were featured with great biosafety, biocompatibility, anti‐swelling, antibacterial adhesion, and blood compatibility. Such properties endowed SCIOPs with great potential for direct incubation with fresh whole blood within certain practical applications like blood sample processing, systematical anticoagulation, blood purification, blood perfusion, and so on.

**Figure 6:**
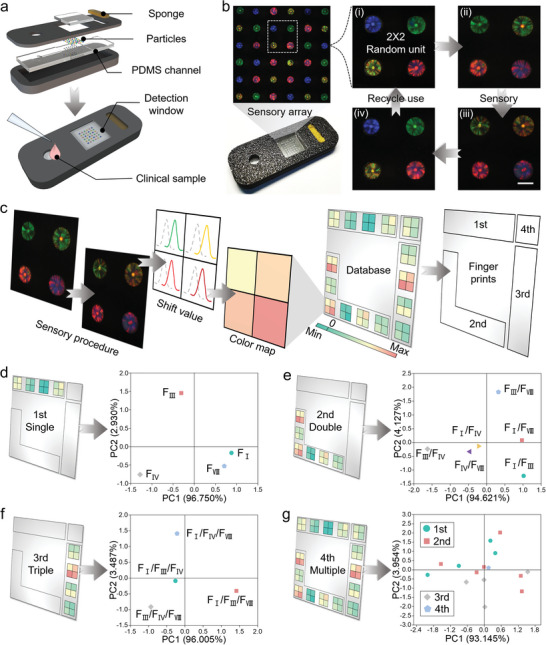
Pattern recognition based on SCIOPs‐integrated POCT strip. a) The scheme of the constructions of the POCT strip. b) The reflection images of sensory arrays are composed of SCIOPs with different structural colors. (i–iv) The optical images of 2 × 2 random sensory units during a complete sensory recycle. c) The schematic illustration of the analysis principle for pattern recognition. d) The PCA score plot of the shift values of the sensory unit to four kinds of single coagulation factors. e) The PCA score plot of the shift values of the sensory unit to six kinds of double coagulation factors combinations. f) The PCA score plot of the shift values of the sensory unit to four kinds of triple coagulation factors combinations. g) The PCA score plot of the shift values of the sensory unit to multiple coagulation factor combinations. The scale bar is 450 µm in (b).

To broaden the applications, a point‐of‐care testing (POCT) strip was constructed, which was integrated with diverse SCIOPs array with different structural colors for simultaneous detection of multiple coagulation factors in plasma samples. The POCT strip was composed of three parts, including a 3D‐printed polylactic acid (PLA) shell, a polydimethylsiloxane (PDMS) channel layer, and a sensory array (**Figure** **6**a; Figures S11 and S12, Supporting Information). A sponge was integrated to drive the liquid sample moving from the inlet to the sensory array with the aid of capillary forces. The sensory array consisted of a group of SCIOPs bearing different structural colors and random distributions. According to the sensory procedure mentioned above, the 2 × 2 unit was randomly selected with corresponding shift values and structural color variations during the complete sensory process (Figure 6b). Additionally, the pattern recognition method was adopted to distinguish plasma samples containing different kinds of coagulation factors according to the characteristics shift values. As schemed in Figure 6c, the shift values of four SCIOPs in random sensory units were measured and transferred to color map. The displacements of chromaticity coordinates of each SCIOP were recorded in the diagram 1931 (Figure S13, Supporting Information). It could be calculated that four coagulation factors could be further combined into 15 groups (single: F_I_, F_III_, F_IV_, F_VIII_; double: F_I_/F_III_, F_I_/F_IV_, F_I_/F_VIII_, F_III_/F_IV_, F_III_/F_VIII_, F_IV_/F_VIII_; triple: F_I_/F_III_/F_IV_, F_I_/F_III_/F_VIII_, F_I_/F_IV_/F_VIII_, F_III_/F_IV_/F_VIII_; multiple: F_I_/F_III_/F_IV_/F_VIII_). The respective color maps of 2 × 2 units appeared obvious differences among the 15 combinations during sensory (Figure S14, Supporting Information), thus these color maps could be considered as individual databases for subsequent pattern recognition. The PCA method was applied to analyze the data and test the recognition efficiency of our sensory unit. As shown in Figure 6d–g, the PCA score plots demonstrated the efficient distinguishment not only between different groups but also within individual groups.

## 3 Conclusion

In conclusion, we reported novel biomimetic SCIOPs for real‐time monitoring of anticoagulation. Based on the template sacrifice method, the SCIOPs were endowed with inverse opal hydrogel skeleton and sufficient specific surface area, which provided binding sites for abundant sulfonic and carboxyl groups to recognize four coagulation factors. The variations of the refractive index and pore sizes during the binding process led to respective shifts of characteristic reflection peak values and structural color. In particular, a complete sensory recycle was achieved due to the SCIOPs could return to initial state after the elution of target molecules, which enabled cyclic utilization and lowered cost. Besides, the biosafety properties of hydrogel components of SCIOPs like biocompatibility, anti‐swelling, antibacterial adhesion, and hemolysis were demonstrated to be excellent. In addition, a POCT strip integrated with a detection array composed of SCIOPs with different structural colors was developed. By analyzing the shift value of each particle, a colorimetric database was established, namely “fingerprint”. The clinical samples containing different combinations of four coagulation factors were distinguished via PCA analysis of the shift values during the sensory process. Finally, proteomics analysis was performed and the results indicated that SCIOPs exhibited similar impact on protein species and abundances of whole blood compared with natural heparin. These features make the SCIOPs highly promising for anticoagulation applications, POCT detection, and clinical disease diagnosis.

## 4 Experimental Section

### Materials

The Silicon dioxide (SiO_2_) nanoparticles with diameters of 220, 235, 255, and 285 nm were self‐synthesized according to the typical Stöber method. Polyethylene glycol diacrylate (PEGDA), acrylic acid (AA), N,N'‐Methylenebisacrylamide (bis), 2‐acrylamido‐2‐methyl‐1‐propanesulfonic acid (AMPS) and 2‐hydroxy‐2‐methyl‐1‐phenyl‐1‐propanone (HMPP) were purchased from Sigma–Aldrich. Hydrofluoric acid (HF), sodium chloride (NaCl), sodium hydroxide (NaOH), calcium chloride (CaCl_2_), nitric acid (HNO_3_), Coagulation Factor I (F_I_), Coagulation Factor III (F_III_) and Coagulation Factor VIII (F_VIII_) were bought from Nanjing WANQING chemical Glass ware&Instrument Co., Ltd. The ELISA kit for C3a, C5a, BK, TAT, and antithrombin‐III were bought from Nanjing YOUMENG Biotechnology Co., Ltd. The heparin‐coated anticoagulated vacuum tubes, ethylene diamine tetraacetic acid‐K_2_ (EDTA‐K_2_)‐coated anticoagulated vacuum tubes, sodium citrate‐coated anticoagulated vacuum tubes, none‐anticoagulation vacuum tubes and disposable venous blood lancet were obtained from Kang Weishi Medical Instrument Co., Ltd. Bacteria *S. aureus* (BNCC133264) was provided by BeNa Culture Collection. The male Sprague‐Dawley (SD) rats were provided by Jinling Hospital, Nanjing, China. All animals were handled strictly according to the recommendations in the Guide for the Care and Use of Laboratory Animals. All animal care and experimental protocols were reviewed and approved by the Animal Investigation Ethics Committee of Jinling Hospital. Distilled water used in all experiments was purified by a Milli‐Q Plus 185 water purification system (Millipore, Bedford, and MA) with a resistivity higher than 18 mΩ•cm.

### Preparation of SCIOPs

The PhCs templates were fabricated based on self‐fabricated glass capillary co‐flow microfluidic device as reported before. First, bis into AA at the mass ratio of 1: 23.5 was dissolved. Then the anticoagulation hydrogel was prepared by mixing 60% (v/v) PEGDA, 15% (v/v) AA, 10% (w/v) AMPS, and 1% (v/v) HMPP. The PhCs templates were totally immersed into such mixture solution for 4 h until the spaces between nanoparticles were filled with pre‐gel solution. The polymerization process was induced under the conditions of UV light for 5s. Subsequently, 40% (v/v) HF was integrated to etch the SiO_2_ nanoparticles for 2 h, and the final SCIOPs were obtained. Then the SCIOPs were immersed in NaOH solutions for 6 h to form sodium salt form. Finally, the particles were washed with deionized water overnight for the removal of excessive NaOH.

### Anticoagulation Test

The SCIOPs‐contained vacuum tube (2 mL) was self‐prepared, where the SCIOPs were previously freeze‐dried and encapsulated into non‐anticoagulation vacuum tubes (0.1 g SCIOPs per tube). Then the tubes were repeatedly vacuumed for 3–5 times until enough negative pressure was formed in the tube. The fresh whole blood was obtained from the postcava of the mice, and the blood was directly extracted into tubes due to the pressure differences. Then the vacuum tube was slightly shaken for 5–10 times to ensure the full contact between whole blood and anticoagulants. The incubation time of the whole blood was set as 5 min. The fresh whole blood was treated with EDTA‐K_2_ and SCIOPs for blood routine tests. For examination of four blood coagulation indexes (APTT, PT, TT, and FIB), the whole blood was initially treated with sodium citrate and SCIOPs, respectively. Then the blood was centrifuged at 1500 rpm for 15 min to separate plasma and blood cells. Such centrifugation process was repeated for three times to totally remove blood cells. The samples for proteomics analysis, complement activation examination, and antithrombin activity measurement were extracted from the whole blood treated with heparin and SCIOPs, respectively.

### Antibacterial Test

The *S. aureus* was chosen for demonstration of the antibacterial adhesion property of the functional hydrogel components. The bacteria were initially stored at −20 °C. The *S. aureus* on the solid medium at 37 °C was cultured overnight. Then the bacteria were suspended with a sterile buffer solution at a turbidity of 0.5 according to McFarland standard. Such bacteria were added on the surface of four groups (culture medium, PEGDA/AA, PEGDA/AMPS, and PEGDA/AA/AMPS) with the same amount. After incubation under the conditions of 37 °C after 12 h, crystal violet staining was used to estimate the antibiofilm effect. In detail, 200 µL crystal violet dye (1 (w/v) %) was added to each group for 5 min, and then excessive dye was removed. After drying at 37 °C, 200 µL acetic acid (33 (v/v) %) was added and incubated for 30 min. Finally, the absorbance intensity was measured at 590 nm for quantitative analysis of antibiofilm properties.

### Fabrication of SCIOPs‐Integrated POCT Strip

The POCT strip was composed of three main layers, including the top layer for observation, the middle layer containing microchannel, and the bottom layer. With the aid of software Solidworks and Auto CAD, the molds were designed with precise dimensional sizes. The top and bottom layers composed of PLA was manufactured by 3D‐printing. PLA was melted at the nozzle position (240 °C) and then cooled down on the platform. The mold was fabricated layer‐by‐layer with the programmed movement of nozzles. PDMS (mixed with curing agent at the weight of 10:1) was used to replicate the structures of the mold and fabricate the middle microchannel layer featured with an array of cylindrical holes. Then the middle layer was treated with oxygen plasma, and the SCIOPs were poured into the cylindrical holes. A glass slide was combined with the middle layer via chip bonding. Finally, the middle layer was placed in the pre‐designed groove of the bottom layer, and the top layer was aligned and fixed with glue. A sponge was added to the rectangle groove and the complete POCT strip was obtained.

### Characterization

The microstructures of template PhCs, the hydrogel‐filled templates, and the SCIOPs were acquired by a field emission scanning electron microscope (FESEM, Ultra Plus, and Zeiss). Optical images of template PhCs, the hydrogel‐filled templates, and the SCIOPs were recorded by a stereoscopic microscope (Jiang Nan) equipped with a CCD camera (Olympus, DP30BW). Reflection spectra were measured by the optical microscope (Olympus, BX51) equipped with a fiber‐optic spectrometer (Ocean Optics, USB2000‐FLG). The UV spectra were collected by the UV–vis spectrophotometer (Cary60, Agilent Technologies, and USA). The FT‐IR spectrum was measured by the Fourier infrared spectrometer (Thermo). The blood routine test and four blood coagulation indexes were achieved by an automatic blood cell analyzer (MEK‐7222K) and an automatic blood clotting analyzer. The proteomics analysis was performed by an ultra‐high resolution biomass spectrometry analysis system (Orbitrap Eclipse).

## Conflict of Interest

The authors declare no conflict of interest.

## Supporting information

Supporting Information

## Data Availability

The data that support the findings of this study are available from the corresponding author upon reasonable request.
