# Supplementary material for: Biomimetic Anticoagulated Porous Particles with Self‐Reporting Structural Colors
Source: Adv Sci (Weinh). 2024 Mar 23;11(22):2400189. doi: 10.1002/advs.202400189 (PMC11165554; doi:10.1002/advs.202400189)
Supplement: Supplementary file 1 — Supporting Information [file ADVS-11-2400189-s001.pdf]

ADVANCED SCIENCE
Open Access

Supporting Information

for *Adv. Sci.*, DOI 10.1002/advs.202400189

Biomimetic Anticoagulated Porous Particles with Self-Reporting Structural Colors

*Hanxu Chen, Feika Bian, Zhiqiang Luo and Yuanjin Zhao**

Copyright WILEY-VCH Verlag GmbH & Co. KGaA, 69469 Weinheim, Germany, 2018.

Supporting Information

Biomimetic anticoagulated porous particles with self-reporting structural colors

*Hanxu Chen, Feika Bian, Zhiqiang Luo, Yuanjin Zhao**

Supporting Figures

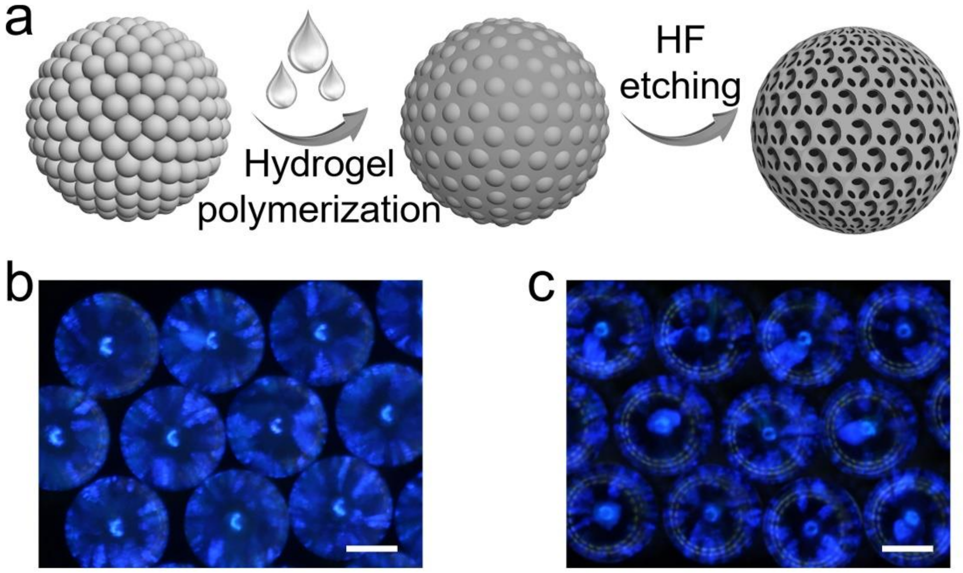

Figure S1. The fabrication procedure of SCIOPs. (a) Scheme of the replication process of PhCs templates, including infiltration of pre-gel solutions, hydrogel polymerization under UV irradiation and HF etching of silicon dioxide nanoparticles. (b) The optical image of PhCs templates bearing blue structural color. (c) The optical image of the templates filled with anticoagulation hydrogel after polymerization. The scale bars are 250 μm in (b) and (c).

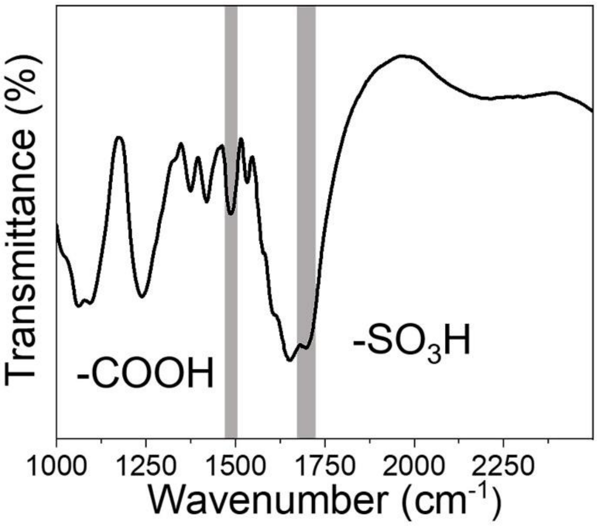

Figure S2. The FT-IR spectrum of heparin sodium. The peaks of carboxyl group (1701.39 cm^{-1}) and sulfonic acid group (1485.59 cm^{-1}) were identified to be similar with the anticogulant hydrogel components of SCIOPs.

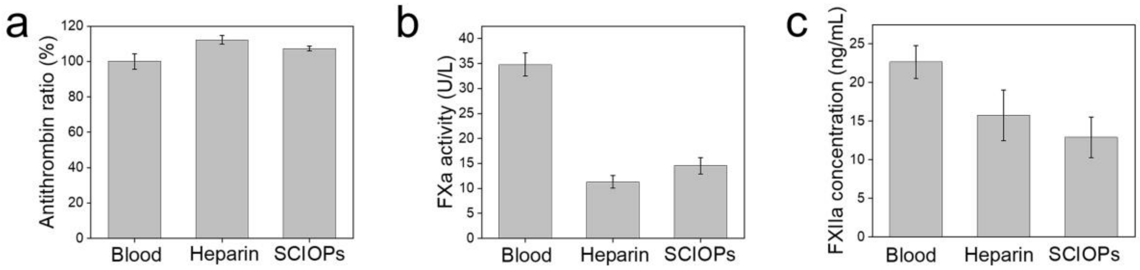

Figure S3. (a) The antithrombin ratio of the blood treated with sodium citrate, heparin and SCIOPs. (b) The activity of FXa of the blood treated with sodium citrate, heparin and SCIOPs. (c) The concentration of FXIIa of the blood treated with sodium citrate, heparin and SCIOPs.

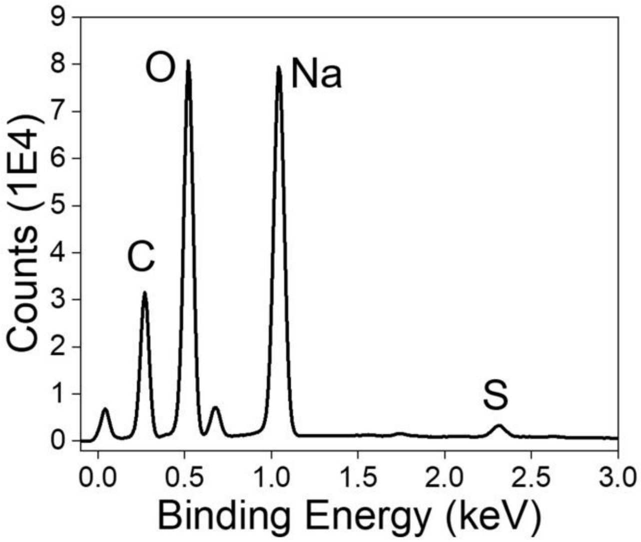

Figure S4. The energy dispersive spectra of the hydrogel components of the SCIOPs. The main elements are C, O, S and Na.

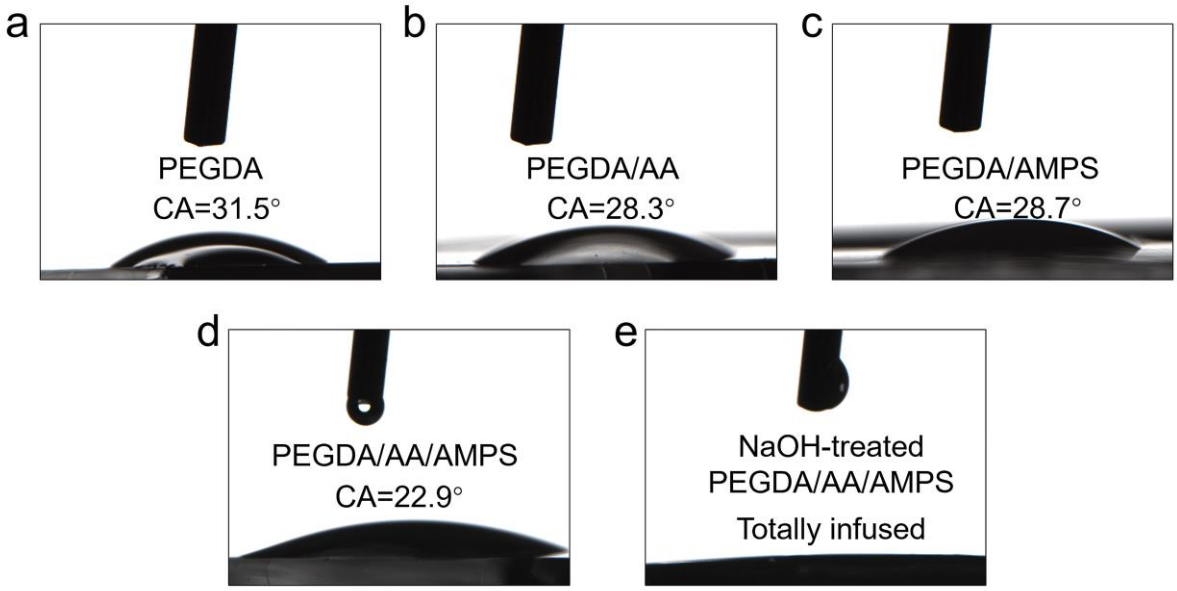

Figure S5. The characterization of the hydrophilic property of the hydrogel component of SCIOPs. The optical image of the water contact angle of the hydrogel components (a) PEGDA, (b) PEGDA/AA, (c) PEGDA/AMPS, (d) PEGDA/AA/AMPS and (e) after NaOH treatment.

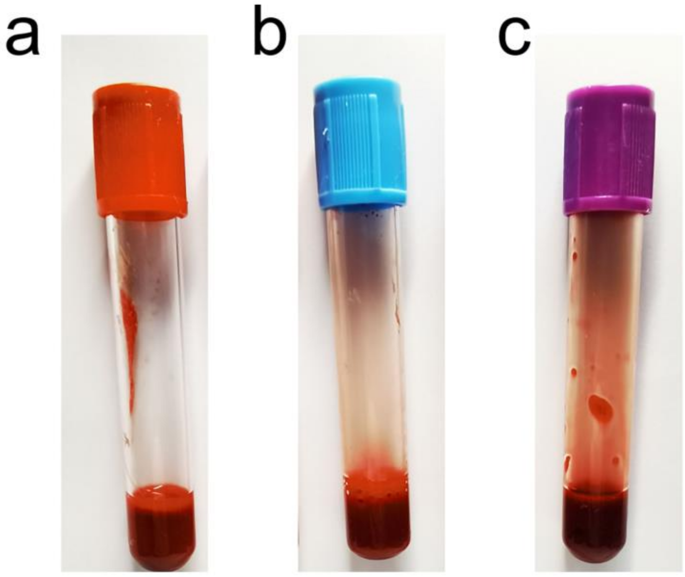

Figure S6. The optical images of the fresh whole blood treated with (a) none anticoagulation, (b) heparin and (c) SCIOPs.

Protein ID	Protein Name	Protein ID	Protein Name	Protein ID	Protein Name	Protein ID	Protein Name
**Q5BJP6**	Ribosome-releasing factor 2	**P02761**	Major urinary protein	**Q63556**	Serine protease inhibitor A3M	**P14272**	Plasma kallikrein
**Q9WTT6**	Guanine deaminase	**Q924S5**	Lon protease homolog, mitochondrial	**P06765**	Platelet factor 4	**P11517**	Hemoglobin subunit beta-2
**P12391**	Neuronal acetylcholine receptor subunit beta-3	**Q6P9V9**	Tubulin alpha-1B chain	**P55797**	Apolipoprotein C-IV	**P06399**	Fibrinogen alpha chain
**P10111**	Peptidyl-prolyl cis-trans isomerase A	**P81828**	Urinary protein 2	**P18424**	Phosphatidylcholine-sterol acyltransferase	**P14480**	Fibrinogen beta chain
**Q62936**	Disks large homolog 3	**P18420**	Proteasome subunit alpha type-1	**Q9JJS8**	Mannan-binding lectin serine protease 2	**P02680**	Fibrinogen gamma chain
**P07808**	Pro-neuropeptide Y	**Q68FS4**	Cytosol aminopeptidase	**P49744**	Thrombospondin-4	**P05544**	Serine protease inhibitor A3L
**P40112**	Proteasome subunit beta type-3	**P22985**	Xanthine dehydrogenase/oxidase	**B0BNN3**	Carbonic anhydrase 1	**P36953**	Afamin
**P35704**	Peroxiredoxin-2	**Q9R0T4**	Cadherin-1	**P27139**	Carbonic anhydrase 2	**P02650**	Apolipoprotein E
**P47967**	Galectin-5	**O08619**	Coagulation factor XIII A chain	**P31394**	Vitamin K-dependent protein C	**P06866**	Haptoglobin
**Q5M889**	Apolipoprotein F	**Q8R2H5**	Phosphatidylinositol-glycan-specific phospholipase D	**Q9WTS8**	Ficolin-1	**P20059**	Hemopexin
**P04797**	Glyceraldehyde-3-phosphate dehydrogenase	**Q64268**	Heparin cofactor 2	**P05371**	Clusterin	**P01946**	Hemoglobin subunit alpha-1/2
**Q63515**	C4b-binding protein beta chain	**P06759**	Apolipoprotein C-III	**P01048**	T-kininogen 1	**P02091**	Hemoglobin subunit beta-1
**P08661**	Mannose-binding protein C	**P01015**	Angiotensinogen	**P02764**	Alpha-1-acid glycoprotein	**P08649**	Complement C4
**Q6L711**	Hyaluronan-binding protein 2	**P60711**	Actin, cytoplasmic 1	**P20762**	Ig gamma-2C chain C region	**Q99PS8**	Histidine-rich glycoprotein
**Q6P6Q2**	Keratin, type II cytoskeletal 5	**P06238**	Alpha-2-macroglobulin	**P19939**	Apolipoprotein C-I	**P05545**	Serine protease inhibitor A3K
**Q6IMF3**	Keratin, type II cytoskeletal 1	**P20759**	Ig gamma-1 chain C region	**Q63514**	C4b-binding protein alpha chain	**P20760**	Ig gamma-2A chain C region
**P01681**	Ig kappa chain V region S211	**Q63207**	Coagulation factor X	**P55159**	Serum paraoxonase/arylesterase 1	**P04937**	Fibronectin
**Q6IFW6**	Keratin, type I cytoskeletal 10	**Q62975**	Protein Z-dependent protease inhibitor	**P04916**	Retinol-binding protein 4		

Table S1. The supporting information of protein ID and protein name for proteomics analysis.

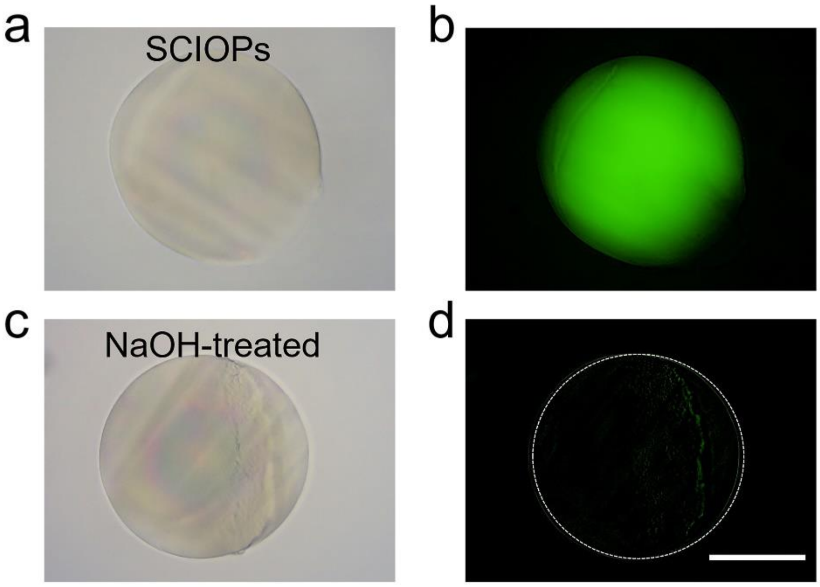

Figure S7. The adsorption of typical plasma protein (bovine serum albumin). (a-b) The bright field and fluorescent images of SCIOPs. (c-d) The bright field and fluorescent images of NaOH-treated SCIOPs. Scale bar is 200 μm.

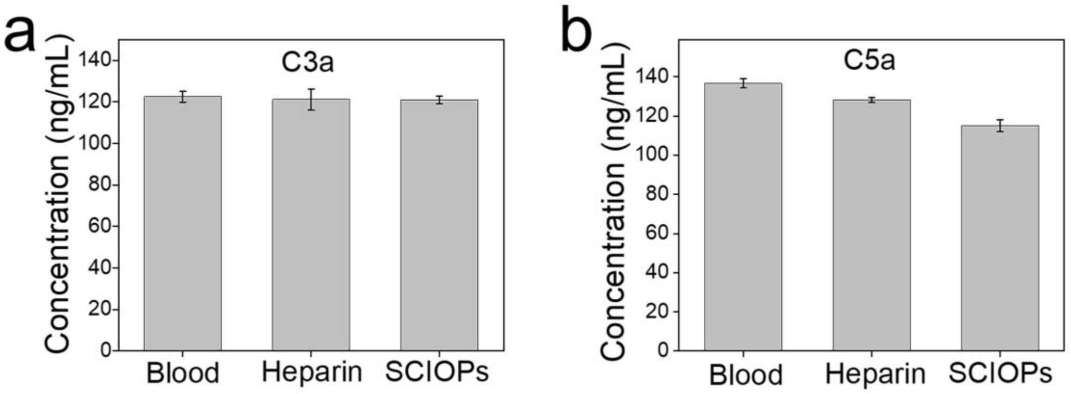

Figure S8. The effects of the SCIOPs on the complement activation system. The C3a (a) and C5a (b) concentration of the blood treated with sodium citrate, heparin and SCIOPs.

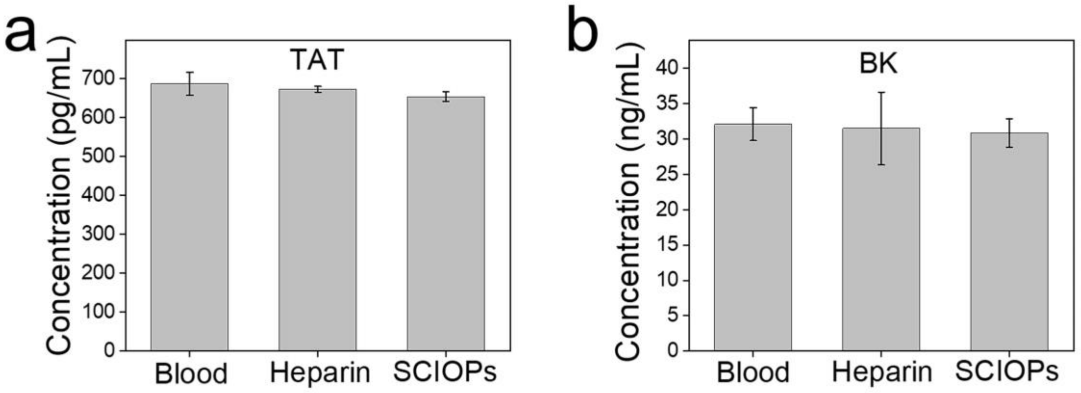

Figure S9. The effects of the SCIOPs on the contact activation system and kallikrein-kinin system. The TAT (a) and BK (b) concentration of the blood treated with sodium citrate, heparin and SCIOPs.

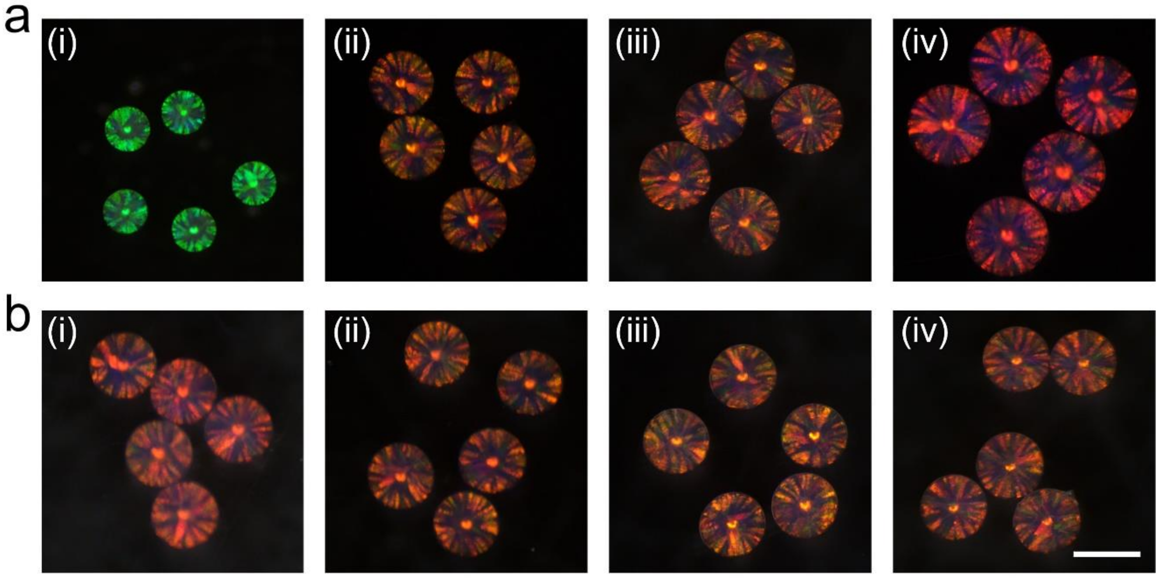

Figure S10. The optical images of SCIOPs in several biofluids. (a) SCIOPs at initial state (i), immersed in NaCl (ii), PBS (iii) and plasma solution (iv). (b) SCIOPs treated with NaOH (i), immersed in NaCl (ii), PBS (iii) and plasma solution (iv). The scale bar is 450 μm.

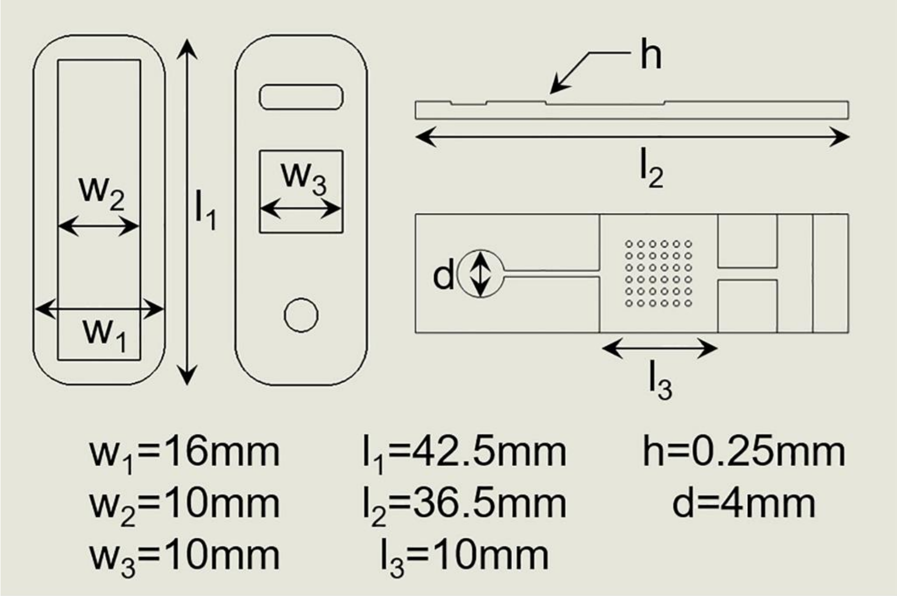

Figure S11. The detailed dimensional drawing of POCT strip.

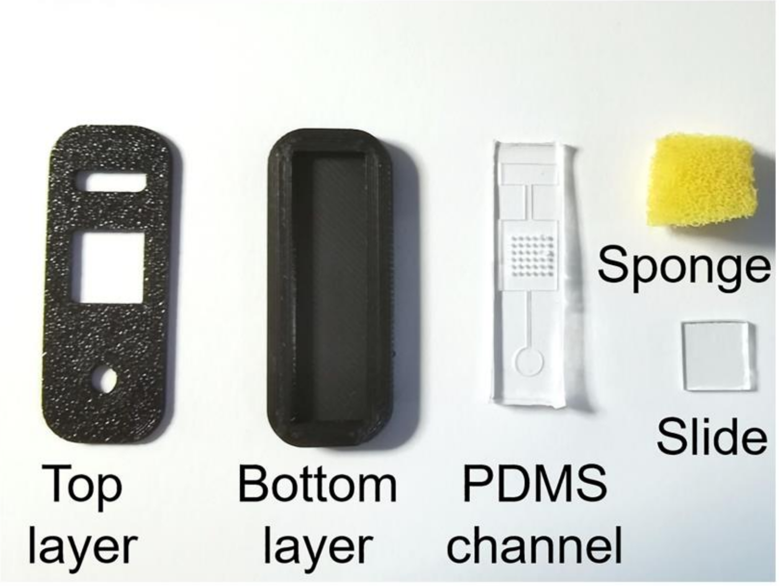

Figure S12. The optical image of the constructions of the POCT strip, including top PLA layer, bottom PLA layer, PDMS channel layer, sponge and slide.

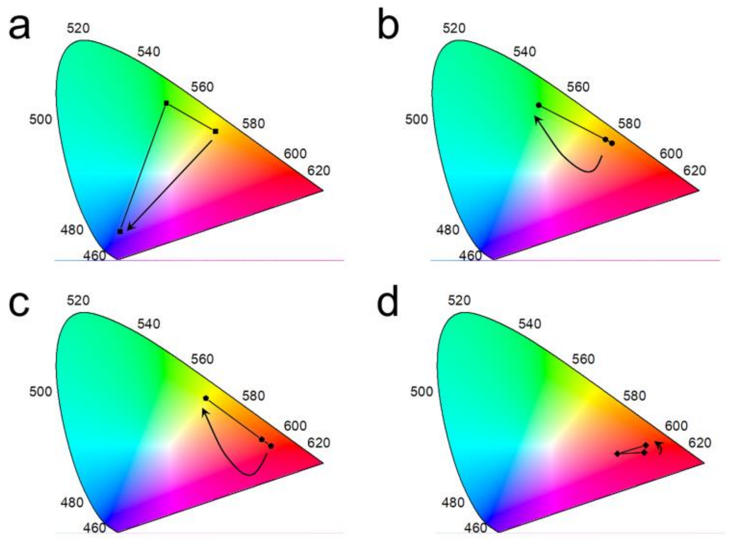

Figure S13. The displacement of chromaticity coordinates of SCIOPs bearing (a) blue, (b) green, (c) yellow, (d) red structural color during a complete sensory recycle in 1931 diagram.

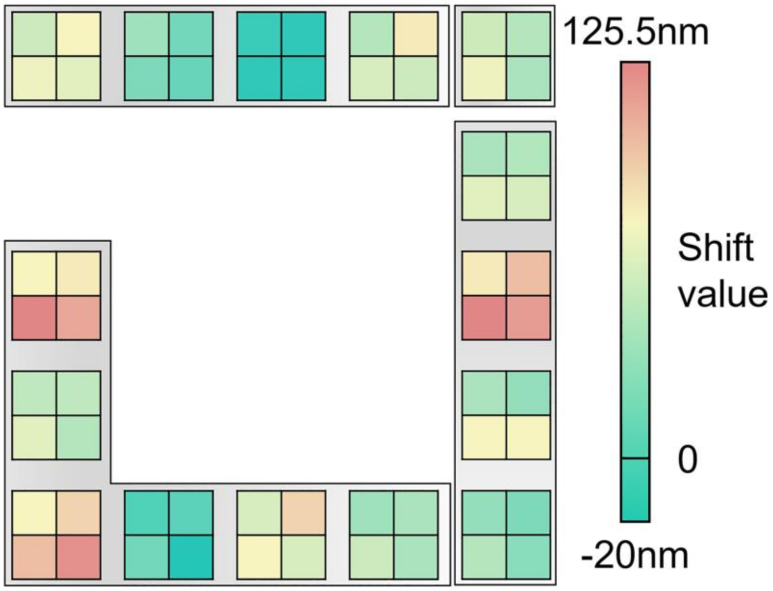

Figure S14. The color map of the shift values of 2x2 detection unit after sensory of 15 kinds of different combinations of four coagulation factors.
